# Proton Leakage Is Sensed by IM30 and Activates IM30-Triggered Membrane Fusion

**DOI:** 10.3390/ijms21124530

**Published:** 2020-06-25

**Authors:** Carmen Siebenaller, Benedikt Junglas, Annika Lehmann, Nadja Hellmann, Dirk Schneider

**Affiliations:** Department of Chemistry, Biochemistry, Johannes Gutenberg University Mainz, 55128 Mainz, Germany; siebenal@uni-mainz.de (C.S.); b.junglas@fz-juelich.de (B.J.); alehma01@students.uni-mainz.de (A.L.); nhellmann@uni-mainz.de (N.H.)

**Keywords:** IM30, Vipp1, membrane fusion, membrane binding, thylakoid membrane, Mg^2+^, pH, CD spectroscopy, quartz crystal microbalance

## Abstract

The inner membrane-associated protein of 30 kDa (IM30) is crucial for the development and maintenance of the thylakoid membrane system in chloroplasts and cyanobacteria. While its exact physiological function still is under debate, it has recently been suggested that IM30 has (at least) a dual function, and the protein is involved in stabilization of the thylakoid membrane as well as in Mg^2+^-dependent membrane fusion. IM30 binds to negatively charged membrane lipids, preferentially at stressed membrane regions where protons potentially leak out from the thylakoid lumen into the chloroplast stroma or the cyanobacterial cytoplasm, respectively. Here we show *in vitro* that IM30 membrane binding, as well as membrane fusion, is strongly increased in acidic environments. This enhanced activity involves a rearrangement of the protein structure. We suggest that this acid-induced transition is part of a mechanism that allows IM30 to (i) sense sites of proton leakage at the thylakoid membrane, to (ii) preferentially bind there, and to (iii) seal leaky membrane regions via membrane fusion processes.

## 1. Introduction

IM30, the Inner Membrane-associated protein of 30 kDa, is conserved in almost all oxygenic photosynthetic organisms, involving cyanobacteria as well as algae and higher plants [1]. Likely, IM30 has evolved via gene duplication from its bacterial ancestor PspA (phage shock protein A) [2], which is suggested to be involved in membrane protection/maintenance in bacteria [3]. In fact, while in bacteria solely PspA is encoded and in algae/plants solely IM30, cyanobacterial genomes typically contain two genes coding for PspA and IM30, respectively [2]. 

Based on computational predictions and secondary structure analyses, IM30 and PspA share a highly similar, mainly α-helical secondary structure [4,5,6,7]. The large, N-terminally localized PspA-domain (~220 aa) consists of six α-helices connected via short linker regions. Based on the x-ray structure of a small PspA fragment (aa 1-144), helices 2 and 3 form an extended coiled–coil structure [8]. An extra C-terminal α-helix (helix 7) is exclusively present in IM30 proteins and probably responsible for the IM30-specific functions [1,2,4,6,9]. Members of the IM30/PspA family, including IM30, PspA, and LiaH, the PspA homolog found in *Bacillus* and *Listeria* species, are known to spontaneously assemble into large, homo-oligomeric ring structures with molecular masses of at least 1 MDa [5,9,10,11,12,13,14,15,16]. Such ring structures have a diameter of 20 nm and a height of 8–11 nm in the case of *E. coli* PspA [5], and a diameter of 25 nm in the case of the *B. subtilis* LiaH [16]. In contrast, IM30 proteins appear to form multiple ring structures (from the same building blocks) [11,14]. IM30 of the cyanobacterium *Synechocystis* sp. PCC 6803 (from here on: *Synechocystis*), the currently best-studied IM30 protein, forms rings with diameters of 24–33 nm, which, however, have a constant height of 13–15 nm [14].

The exact physiological function of IM30 is still not finally clarified (reviewed in detail in [17]), albeit two major functions have emerged in recent years: A membrane-stabilizing/protecting function and a membrane remodeling function (recently reviewed in [18]).

IM30 likely shares its membrane protecting function with PspA, its bacterial ancestor. PspA is known to stabilize membranes, which is required to maintain the proton motive force (PMF) in many Gram-negative bacteria [3,19,20]. Both, PspA and IM30 bind to negatively charged lipid membranes in a curvature-dependent manner [13,19,21,22], and IM30 can even replace PspA in organisms where PspA was deleted, but not vice versa [23,24]. In chloroplasts and cyanobacteria, maintaining the membrane integrity and organization is especially crucial for preserving an electrochemical gradient across the thylakoid membrane (TM) system. The TM system is continuously stressed and impaired due to oxidative damage caused by reactive oxygen species generated in the photosynthetic electron-transfer reaction [25]. Especially highly curvature-stressed TM regions could bear the risk of dissipating the ∆pH across the TM, as lipid packing defects clearly are enhanced with increasing membrane curvature [26,27]. In fact, recent *in vivo* studies show that IM30 particularly localizes to highly curved TM margins under light-stress conditions, where it forms large clusters [28]. These clusters potentially represent protective IM30 assemblies on the membrane [18,26,29]. While the molecular details of its proposed membrane-protecting activity are rather enigmatic, it has been shown that membrane binding of IM30 increases the membrane lipid order, which might be key for membrane protection [13].

Besides membrane protection, IM30 also has a membrane remodeling activity. Photosynthetic organisms continuously rearrange their TM system to dynamically adapt their photosynthetic apparatus to changing light intensities [30,31,32,33,34]. TM membrane rearrangements involve membrane fusion events [35], which are potentially mediated by IM30 [21]. In fact, IM30 can fuse TM-mimicking model membranes *in vitro* when Mg^2+^ is present [21,36]. Direct binding of Mg^2+^ to IM30 induces structural changes, such as increased exposure of hydrophobic surface regions, an overall more compact structure and increased protein stability [37]. The Mg^2+^ concentration in the cyanobacterial cytoplasm and the chloroplast stroma is light-dependent and is related to the photosynthetic activity of chloroplasts and cyanobacteria [38], which directly links IM30-mediated membrane fusion processes to photosynthetic electron-transfer reactions.

As IM30 is predominately found at TM regions that are prone to membrane defects, such as the highly curved TM margins [28], IM30 potentially seals membrane regions where protons leak out of the TM lumen, and thus, IM30 likely encounters conditions with significantly lowered (local) pH. Therefore, in the present study we have analyzed the structural properties and membrane interaction of the *Synechocystis* IM30 under acidic conditions. 

We here show that IM30 adopts an altered structure and that membrane binding and membrane fusion of IM30 is enhanced at around pH 5. We propose that the structural rearrangements of IM30 induced by an acidified environment enable the protein to specifically sense and bind to defective TM regions where protons leak out of the TM lumen into the cytoplasm, which increases the membrane fusion capabilities of IM30.

## 2. Results

### 2.1. IM30 Has an Unconventional Secondary Structure at pH 5.5

**Figure 1:**
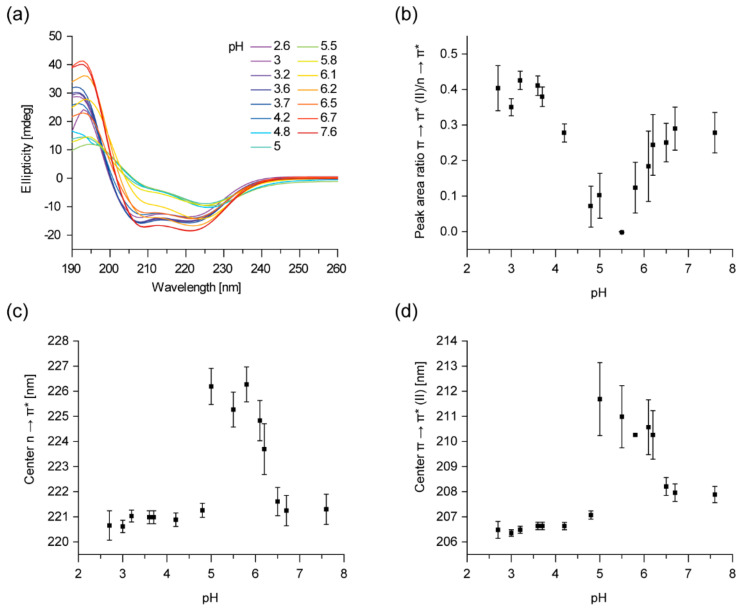
The secondary structure of IM30 (the Inner Membrane-associated protein of 30 kDa) changes in an acidic environment. (**a**) Far UV CD-spectra of IM30 were measured in a pH range from 7.6 to 2.6. The spectrum changes from a typical α-helix spectrum to an atypical CD-spectrum at around pH 5.5 and back to a “typical” α-helix spectrum below pH 4. The spectra were averaged from three samples, no error bars are shown. (**b**) The ratio of the *n* → π* transition peak area and of the parallel polarized π → π* (||) transition peak is shown in dependence on the pH. A minimal peak area ratio can be observed at ~pH 5.5 (*n* = 3, error bars=SD). (**c**) The dependence of the *n* → π* transition peak center on pH reveals a maximum of the peak shift at ~pH 5.5 (*n* = 3, error bars=SD). (**d**) The dependence of the peak center of the parallel polarized π → π* (||) transition reveals a maximum of the peak shift at ~pH 5.5 (*n* = 3, error bars=SD).

Based on sequence analyses, IM30′s secondary structure is roughly 80% α-helical with seven predicted α-helices that are connected via flexible linkers [6,12,14,37,39]. In line with the prediction, the far-UV CD spectrum of IM30 at physiological pH (pH 7.6) shows pronounced minima at 222 (*n* → π* transition peak) and 208 nm (π → π* (||) transition peak) (Figure 1a), as typical for α-helices. Yet, when the pH was successively decreased from 7.6 to 5.5, the CD-spectrum of IM30 changed drastically (Figure 1a): the amplitude of both transitions strongly decreased with a minimum at around pH 5.5, but the amplitude of the π → π* (||) transition peak dropped more dramatically. As a representation for the spectral shape change, the ratio of the peak areas of the two transitions is shown in Figure 1b. The area ratio found at pH 7.6 (~0.3) drops to ~0 at pH 5.5, and thus, the π → π* (||) transition does barely contribute to the CD spectrum at this pH. When the pH was further decreased below 5.5, the structural changes vanished steadily, again reaching an area ratio of 0.3 at pH 4 and even increasing slightly at pH 3.5 to 2.6, compared to pH 7.6 (Figure 1b).

Additionally, the *n* → π* transition peak shifted from ~222 nm at pH 7.6 to ~226 nm (Figure 1c), and the π → π* (||) transition peak from ~208 nm at pH 7.6 to ~211 at around pH 5.5 (Figure 1d). Lowering the pH beyond 5.5 reversed the described effect, and below pH 5.0, the center of the *n* → π* transition peak was again located at ~221 nm and the π → π* (||) transition peak centered at ~207 nm.

Thus, at around pH 5.5, the CD-spectrum of IM30 significantly differs from the spectrum measured at physiological pH, indicating a pronounced change of the secondary structure. Unfortunately, we did not succeed in categorizing the IM30 secondary structure at pH 5.5 based on a superposition of canonical secondary structure elements, *i.e.*, α-helix, β-sheet or random-coil. Thus, the protein may contain a large fraction of atypical secondary structure elements at this pH (such as β-turns, as determined by the BeStSel algorithm [40]). The spectrum to some extent resembles a mainly α-helical spectrum, distorted by absorption flattening due to light scattering [41,42]. However, the photomultiplier voltage did not show any suspicious, pH-dependent increase. Possibly, at least partially atypical β-sheets are formed, as seen in some lectins, where the β-sheet characteristic minimum is shifted from 217 to about 225 nm [43]. Interestingly, below pH ~ 4 and even at pH values as low as pH 2.6, IM30 again shows a CD-spectrum typical for regular α-helices (Figure 1a).

Taken together, the overall structure of IM30 is highly pH-dependent, and IM30 adopts an uncommon secondary structure at a mildly acidic pH of 5.5.

### 2.2. The Tertiary Structure of IM30 Changes upon Acidification

**Figure 2:**
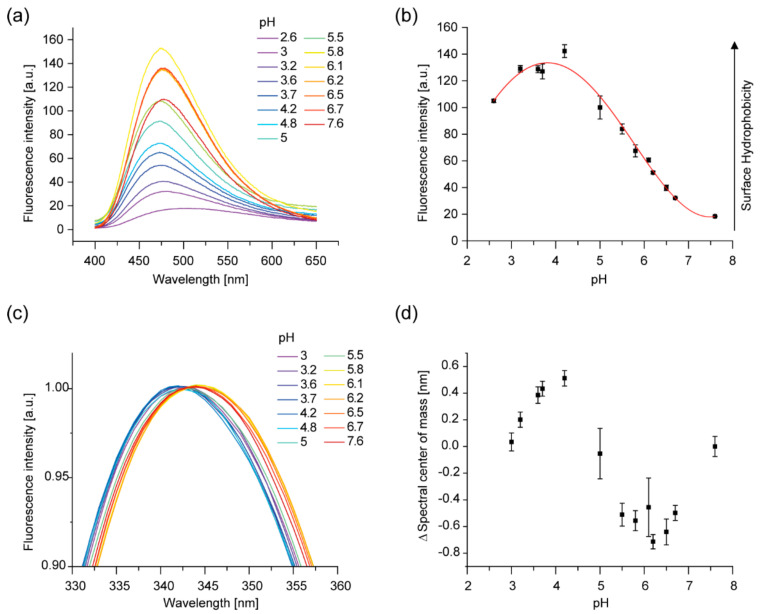
The tertiary structure and surface properties of IM30 change in an acidic environment. (**a**) 8-anilinonaphthalene-1-sulfonic acid (ANS) fluorescence spectra of IM30 were measured at decreasing pH. At lowered pH, an increase of the fluorescence intensity and a blue shift was observed. The spectra shown are the average of three samples; no error bars are shown. (**b**) The ANS fluorescence intensity at 490 nm changes with pH, exhibiting a maximum at ~pH 4.5 (*n* = 3, error bars=SD). (**c**) Intrinsic Trp fluorescence spectra suggest an altered environment of the single IM30 Trp residue upon decreasing the pH from pH 7.6 to 3. The spectra are normalized at 343 nm and smoothed using a Savitzky–Golay-filter (*n* = 3, no error bars shown). (**d**) To quantify the differences between the measured Trp spectra, the spectral center of mass was calculated. A biphasic transition upon acidification, with a minimum at ~pH 6 and a maximum at ~pH 4.5, is observed (*n* = 3, error bars=SD).

The changes in the secondary structure described above are accompanied by a clear increase of the exposed hydrophobic area, as monitored by the spectral changes of the 8-anilinonaphthalene-1-sulfonic acid (ANS) fluorescence (Figure 2a). ANS is a fluorescent dye that changes its spectral properties upon binding to hydrophobic protein surfaces via its anilino-naphthalene group. Free ANS has an emission maximum at 525 nm, which blue shifts in a more hydrophobic environment. This spectral shift is accompanied by a pronounced intensity increase [44]. The fluorescence intensity of ANS in the presence of IM30 steadily increased with decreasing pH, reaching a maximum at pH ~4.5, followed by a shallow decrease (Figure 2b), accompanied by the characteristic blue shift of the emission maximum. In contrast, the fluorescent properties of ANS in absence of IM30 were not affected by the pH (Supplementary Figure S1). 

Thus, also the tertiary structure of IM30 clearly changes upon acidification, resulting in an increased exposure of hydrophobic surfaces. Next, we probed whether the environment of the single Trp of *Synechocystis* IM30, located within the extended coiled-coil formed by helices 2 and 3 [14], is also altered. While no dramatic effect on the Trp’s fluorescence emission spectrum was observed (Supplementary Figure S2, Figure 2c), analysis of the spectral shape based on the spectral center of mass revealed also a biphasic trend, with a minimum at around pH 6 and a maximum at about pH 4 (Figure 2d). Note that the Trp fluorescence intensity is in general not pH-dependent upon acidification to a pH as low as 3, whereas further lowering the pH leads to quenching of the Trp fluorescence [45].

Thus, the coiled–coil region appears to rearrange to some extent, leading to a slightly more hydrophobic environment at mildly acidic pH, followed by a slight increase in polarity at further lowered pH values.

In conclusion, the Trp71 solvent exposure appears to be a biphasic process when the pH decreases. A similar effect, with a maximum at around pH 4.5, has been observed for the surface hydrophobicity of IM30 when IM30 was transferred from pH 7.6 to 2.6. These results, combined with the observed changes of the IM30 secondary structure (Figure 1), clearly suggest a rearrangement of the IM30 protein structure upon acidification.

### 2.3. Acidic pH and Mg^2+^ Have Similar Effects on the IM30 Structure

Mg^2+^ binds directly to IM30, which causes several secondary structure rearrangements, an increased surface hydrophobicity as well as an overall more compact protein structure [37]. Indeed, the Mg^2+^-induced changes in IM30′s CD spectrum are similar to the pH-induced changes observed here. In particular, in presence of Mg^2+^ the amplitude of the π → π* (||) transition is also decreased compared to the *n* → π* transition. Yet, the effects observed upon Mg^2+-^binding were not as pronounced as observed here upon lowering the pH. Furthermore, a shift of the *n* → π* transition peak was not observed at constant pH in absence vs. presence of Mg^2+^.

**Figure 3:**
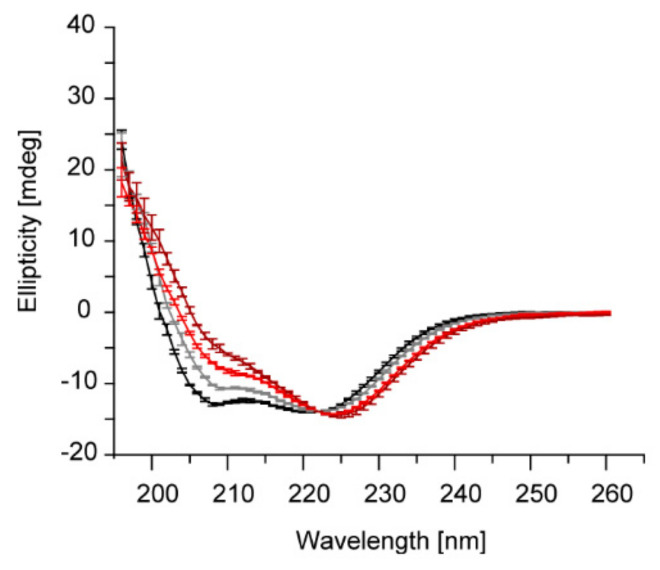
Acidic pH and Mg^2+^ increase the 222/208 nm ratio. Far UV CD-spectra of IM30 measured in absence (black), in presence of Mg^2+^ (gray) at pH 7.6, and of IM30 in absence (dark red) and in presence of Mg^2+^ (light red) at pH 5.5. Spectra were normalized at 222 nm (*n* = 3, error bars=SD).

Thus, we next tested whether Mg^2+^ still binds to IM30 at pH 5.5 and whether the effect on the protein structure observed upon addition of Mg^2+^ is additive at pH 5.5, *i.e.*, would cause a more intense decrease in the amplitude of the *n* → π* transition peak. Yet, under these conditions we observed a slight increase of the amplitude (Figure 3). However, Mg^2+^ clearly binds to IM30 also at more acidic pH and induces a change in the IM30 structure. Interestingly, the CD-spectra of IM30 measured at pH 7.6 and 5.5 are very similar when Mg^2+^ is present (Figure 3), indicating that Mg^2+^-binding potentially stabilizes a particular IM30 structure.

### 2.4. The Membrane-Binding Affinity of IM30 is Increased at Low pH

Interaction of IM30 with membrane surfaces is essential for its proposed *in vivo* functions, involving membrane protection and membrane remodeling [18,21,23,26,28,46,47,48]. To assess the impact of a decreased pH on the membrane-binding propensity of IM30, we used two different quantitative experimental setups.

**Figure 4:**
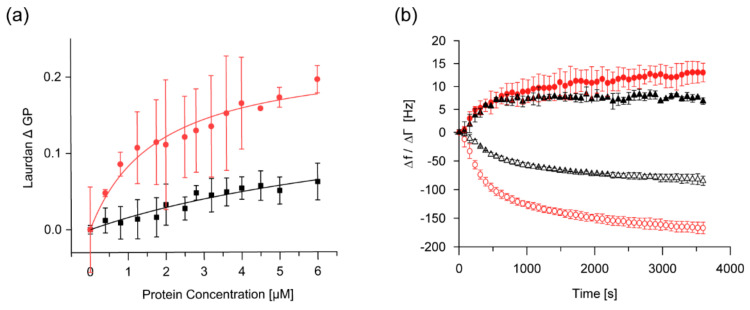
IM30 membrane binding is enhanced at low pH. (**a**) Steady-state membrane binding of IM30 was measured using Laurdan fluorescence spectroscopy at pH 5.5 (red) and pH 7.6 (black). The Laurdan ΔGP at 100 µM lipid (40% DOPG/60% MGDG) with 0–6 µM IM30 was strongly increased at pH 5.5. The membrane-binding affinity of IM30 at pH 7.6 is characterized by a K_d_ = 9.8 ± 5.8, and at pH 5.5 by a K_d_ = 1.8 ± 0.4 (*n* = 3, error bars=SD). (**b**) Kinetics of IM30 binding (4.5 µM) to a solid-supported bilayer (20% DOPG/80% DOPC) was monitored using QCM-D at pH 5.5 (red) and pH 7.6 (black), respectively. Empty symbols represent the change in frequency (∆f), filled symbols represent the change in damping (∆Г). At pH 5.5, faster binding kinetics reflected by the change in frequency Δf was observed (*n* = 3, error bars=SD).

We first monitored changes in the Laurdan fluorescence spectra upon addition of protein, as described previously [13,21]. As a mimic of a TM-membrane we used liposomes containing 40% of the negatively charged lipid DOPG and 60% of the most abundant TM lipid MGDG [49], and compared the changes in Laurdan’s GP value induced by IM30 binding at pH 7.6 and pH 5.5, where the secondary structure changes were maximal. While the Laurdan fluorescence spectra monitored in absence of IM30 did not change when the pH was decreased from 7.6 to 5.5 (Supplementary Figure S3), binding of IM30 to DOPG/MGDG membranes clearly differed at pH 5.5 compared to pH 7.6, as quantified by comparing the GP values (Figure 4a). Compared to pH 7.6, the amplitude of the change in GP is substantially larger at pH 5.5, accompanied by a steeper increase at low protein concentrations, indicating a higher membrane-binding affinity or an increased extent of polarity changes per bound IM30. The *K_d_* extracted from these curves was calculated to be ~ 9.8 ± 5.8 µM at pH 7.6. A substantially lower *K_d_* of ~ 1.8 ± 0.4 µM was determined at pH 5.5. Thus, the IM30 membrane binding affinity clearly is increased at lowered pH.

To further support the observations and interpretations of the Laurdan spectral changes and to further analyze membrane-binding of IM30 at acidic pH, we next analyzed binding of IM30 to a solid supported lipid bilayer (SLB) employing a quartz-crystal microbalance (QCM) (Figure 4b). Due to difficulties in formation of SLBs with a high content of anionic lipids, we were limited to SLBs containing 80% DOPC and 20% DOPG in our QCM measurements. In QCM measurements, mass deposition on a quartz-chip is followed by monitoring the decrease in resonance frequency of the chip. If the adsorbed layer is rigid, the observed frequency shift is directly proportional to the mass adsorbed on the chip, and thus the mass can be calculated [50,51]. However, if the layer is viscoelastic, as indicated by a visible change in damping of the oscillation, the frequency shift is reduced, and the calculation of the mass based on a rigid layer leads to an underestimation of the adsorbed mass (“missing mass effect”) [52].

Binding of IM30 to the SLB at pH 7.6 was observed as a decrease of the resonance frequency over a time course of 3500 s (Figure 4b), accompanied by an increase in the damping signal. Interestingly, the changes in the frequency and damping were substantially larger at pH 5.5, reaching a final level of ~ ∆f = −150 Hz and ∆Г = 12 Hz after 1000 s, compared to −75 Hz and 7 Hz at pH 7.6. Since both frequency and damping differ by about the same factor, we conclude that at pH 5.5 more IM30 binds to the SLB, in excellent agreement with the increased membrane-binding affinity observed with Laurdan-labeled MGDG/DOPG liposomes (Figure 4a). 

### 2.5. IM30-Mediated Membrane Fusion is Enhanced at Low pH

IM30 triggers the fusion of TM-mimicking model membranes, which is strictly Mg^2+^ dependent [21,36,53]. As we observed changes of the IM30 structure at acidic pH that appear to be related to the structural changes caused by Mg^2+^ binding [37], we wondered whether the membrane fusion activity of IM30 is also pH-dependent.

**Figure 5:**
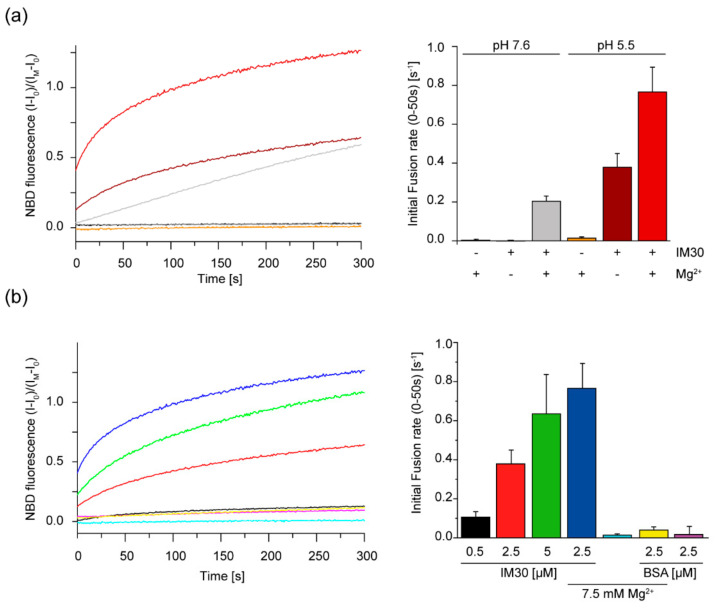
IM30-mediated membrane fusion is enhanced under acidic conditions. (**a**) Liposome fusion was measured in presence of 2.5 µM IM30 and/or 7.5 mM Mg^2+^ by a FRET-based fusion assay. No membrane fusion was observed in presence of solely 7.5 mM Mg^2+^ at pH 5.5 or pH 7.6, respectively. At pH 5.5 and in presence of 7.5 mM Mg^2+^, addition of 2.5 µM IM30 triggered increased membrane fusion compared to pH 7.6. Notably, at pH 5.5, membrane fusion was observed even in absence of Mg^2+^. The initial fusion rates were calculated from the first 50 s. The curves represent the average of three independent measurements (*n* = 3, error bars=SD). The colors of the fusion curves (left) are defined in the bar diagram (right). (**b**) Increased liposome fusion was measured at pH 5.5 when the IM30 content was increased from 0.5 - 5 µM. Addition of 7.5 mM Mg^2+^ further enhanced the membrane fusion rate. Controls containing BSA instead of IM30 did not show any membrane fusion activity. The curves represent the average of three independent measurements. The initial fusion rate in the first 50 s was calculated for membrane fusion at pH 5.5 in presence of 0.5–5 µM µM IM30, for 2.5 µM IM30 + 7.5 mM Mg^2+^ and for the corresponding controls using BSA instead of IM30 (*n* = 3, error bars=SD). The colors of the fusion curves (left) correspond to the bar diagram (right).

Since sufficiently high concentrations of Mg^2+^ alone result in fusion of liposomes containing anionic lipids [21], we first analyzed the IM30-independent fusion propensities of the DOPG/MGDG (40/60) liposomes at different pH values in presence of 15 mM Mg^2+^. Here, we observed a decreased fusion when the pH was lowered from 7.6 to 5.5 when 15 mM Mg^2+^ was present (Supplementary Figure S4), and no fusion was observed at 7.5 mM Mg^2+^. In stark contrast, the IM30-induced membrane fusion activity in presence of 7.5 mM Mg^2+^ was substantially higher at pH 5.5 than at pH 7.6 (Figure 5a). Interestingly, IM30 was able to induce membrane fusion even in the absence of Mg^2+^ at pH 5.5 (Figure 5a,b). However, addition of 7.5 mM Mg^2+^ to 2.5 µM IM30 further increased its membrane fusion activity, outperforming the activity of 5 µM IM30 in absence of Mg^2+^. Thus, Mg^2+^ clearly supports, but is not mandatory for, IM30-mediated membrane fusion at pH 5.5.

## 3. Discussion

### 3.1. The Structure and Membrane Interaction of IM30 is Modulated by a More Acidic pH

We here show that a more acidic environment induces changes in the IM30 secondary and tertiary structure (Figure 1 and Figure 2). While all observed changes were at least biphasic, not all followed the exact same pattern in the pH range between 7.6 and 2.6. The surface hydrophobicity changed almost linearly between pH 6.7 and pH 4.2, as indicated by the increase in ANS fluorescence, whereas the changes in the CD-spectrum are most pronounced at pH 5.5, and have regained basically the original shape at pH 4.0. Thus, the protein structure observed at around pH 5.5 further alters at pH values below 5.5, most likely due to further protonation of particular amino acid side chains. While it remains unclear what structure is actually acquired at pH 5.5, the protein potentially contains some unconventional β-sheet structures, as, for example, described in McCubbin et al. [54]. Similar CD-spectra with a shifted β-sheet minimum were, for example, observed when the structure of small peptides was monitored upon pH changes or TFE treatment [55]. The intimate environment of Trp71 also changed in a biphasic manner when the pH was lowered, indicating a structural modification involving the region containing the coiled-coil forming helices 2 and 3 (Figure 2). Nevertheless, the observed rather minor changes of the Trp fluorescence indicate no drastic changes of the protein’s tertiary (and potentially quaternary) structure, such as protein denaturation.

The observed changes of the secondary structure (Figure 1 and Figure 3) and the increase in the surface hydrophobicity (Figure 2) observed at low pH values, resemble structural changes observed previously in presence of Mg^2+^ [37]. Since presence of Mg^2+^ is mandatory for IM30′s fusion activity (at physiological pH), we investigated to what extent an acidic pH influences the IM30 fusion activity. Indeed, when lowering the pH from pH 7.6 to 5.5, IM30-mediated membrane fusion was dramatically enhanced (Figure 5), which was not observed for Mg^2+^-induced membrane fusion (Supplementary Figure S4). Furthermore, at pH 5.5, membrane fusion was triggered by IM30 even in the absence of Mg^2+^, showing that IM30 is already fusion-competent under these conditions (Figure 5). This implies that the alterations in the CD spectrum observed at this pH are not only similar to the ones observed in presence of Mg^2+^, but also that possibly the underlying structural changes and thus the functional consequences are similar. Thus, apparently both, protons and Mg^2+^, induce and/or stabilize a fusion-competent conformation of IM30. As the divalent cation Mg^2+^ binds to IM30, potentially via negatively charged amino acid side-chains, protonation of negatively charged amino acids of IM30 is not unexpected and appears to have similar effects as Mg^2+^-binding. However, protons appear to be much more effective in this aspect, since the structural changes are considerably more pronounced upon lowering the pH than upon addition of Mg^2+^. 

The enhanced fusion activity observed at acidic pH might be related to some extent to the increased IM30 membrane-binding propensity, as observed via Laurdan fluorescence spectroscopy as well as via QCM measurements. While membrane binding of IM30 is usually attributed to electrostatic interactions with negatively charged lipids [21], our results here indicate a partial contribution of hydrophobic interactions to membrane binding. The increased proton concentration at lowered pH induces increased exposure of hydrophobic patches (Figure 2), thereby promoting hydrophobic interactions, leading to a higher membrane-binding affinity. On the other hand, increased proton concentrations will lead to a reduction of the membrane surface charge density, due to protonation of the lipid PG. Thus, it seems that the exposure of hydrophobic surface area accompanying proton binding outweighs the weakened electrostatic interactions between IM30 and the negatively charged membrane.

### 3.2. Physiological Implications of a pH-Responsive IM30 Structure and Function

Proteins of the PspA/IM30/LiaH family are involved in membrane stabilization in bacteria and chloroplasts [29]. At least in *E. coli* and *Yersinia enterocolitica,* membrane defects and secretin mislocalization are sensed by the membrane proteins PspB and C, which induce the *phage shock protein (psp)* stress response via binding the effector protein PspA at the damaged membrane site, where it can stabilize the membrane [56]. Yet, not all bacteria expressing *pspA* encode the full *psp* response system: *e.g.*, in *Streptomyces lividans* the *psp* system only consists of a PspA homolog, which is also crucial under stress conditions [57], giving rise to the assumption that not all PspA homologs require PspB and PspC for being guided to membrane defects. The same appears to be true for IM30, as no IM30-related PspB or PspC homologs have been identified in cyanobacteria or chloroplasts yet. However, it still is enigmatic how PspA and IM30 ultimately stabilize membranes. 

While PspA is suggested to maintain the proton motive force across the cytoplasmic membrane in a large number of Gram-negative bacteria [1,3,19,56], IM30 is thought to be of special importance for stabilizing TMs in chloroplasts and cyanobacteria [1,46,48]. Maintaining a ∆pH across the TM network in cyanobacteria as well as in chloroplasts is vital for energy production. In cyanobacteria, the TM system is a completely separated compartment [58], and due to proton translocation driven by electron transfer reactions, the pH of the thylakoid lumen is much more acidic than the pH of the cytoplasm. In the dark and at an external pH of about 7, the pH of the cyanobacterial cytoplasm is between 7 and 8, whereas the pH of the thylakoid lumen is between 4.5 and 5.5 [59,60,61,62,63,64]. In the light, the pH increases by at least 0.5 pH units in the cytoplasm, while the pH in the thylakoid lumen also roughly decreases by 0.5 pH units, due to the uptake of protons into the thylakoid lumen and extrusion of protons into the surrounding medium [65,66].

Cyanobacteria and chloroplasts need to constantly maintain a stable proton gradient across the TM, which involves preserving the integrity of a TM system. However, due to curvature stress and light-induced oxidative stress the TM system is prone to get destabilized or even damaged. In fact, IM30 appears to be of special importance for stabilizing the TM system in chloroplasts and cyanobacteria [1,46,48]. We here show that the structure of IM30 is pH sensitive, with a maximum effect on the IM30 structure observed in the range of pH 4 to 5 (Figure 1 and 2). Yet, IM30 is localized in the chloroplast stroma and cyanobacterial cytoplasm, respectively, with a fraction bound to the cytoplasmic face of the TM and the cytoplasmic membrane or inner envelope membrane in cyanobacteria or chloroplasts, respectively [28,67]. Thus, IM30 typically experiences pH values between 7 to 8.

However, defects in the TM system will result in protons leaking out of the TM lumen into the cytoplasmic space, leading to a spatio-temporal accumulation of protons in close proximity to the leak. We here suggest that IM30 “senses” and “repairs” such TM defects by changing its structure if located nearby areas of the TM where the proton concentration is locally increased. Although we cannot yet provide a detailed molecular mechanism, we show here that a decreased pH results in rearrangements of the IM30 structure (Figure 1 and Figure 2). Most likely, acidification leads to protonation of ionizable amino acid side chains, which likely affects intra- and intermolecular electrostatic interactions as well as the protein’s net surface charge [68]. The responsible pH-sensitive amino acids are hard to predict without a crystal structure of the protein, especially considering potential large pKa shifts of (buried) amino acids in proteins [68]. Nevertheless, the observed structural alterations clearly enhance the IM30 membrane binding affinity (Figure 4), which could result in preferential binding of IM30 to defect TM regions. How exactly IM30 stabilizes the defect membrane regions upon binding is currently unclear. Accumulation of IM30 at defined membrane regions has been observed *in vivo*, and it has been hypothesized that IM30 (as well as PspA) rings disassemble on membrane surfaces to form membrane-protective clusters [18,26,29]. However, experimental evidence supporting (or challenging) this hypothesis is missing thus far.

We here additionally show that the membrane fusion activity of IM30 is strongly enhanced at pH 5.5, even in the absence of Mg^2+^ (Figure 5). While it is reasonable to expect that an increased membrane-binding affinity results in an increased membrane fusion activity of IM30, membrane fusion and membrane protection would actually contradict each other at first glance, as membrane fusion requires partial membrane destabilization.

Then why should the IM30 membrane fusion activity get “activated” at defective membrane regions? We suggest that membrane sealing/repair at these defective regions involves membrane fusion. At pre-destabilized membrane regions, the energy cost for lipid reorientation is significantly lowered. Thus, it is reasonable to expect an increased fusion activity of IM30 especially at these sites, where IM30 is crucially involved in sealing the membrane leak. Membrane fusion might function as an active membrane repair mechanism: IM30 binds to defective and/or stressed membrane regions and fuses an intact membrane patch to this area to seal the defect. Nevertheless, the general mechanism of TM repair *in vivo* still is largely unknown. Yet, a similar physiological role has *e.g.*, been suggested for the fusion activity of the dynamin-like protein DynA in *B. subtilis*, when cells are stressed by pore-forming agents, such as nisin or phages [69,70]. Similar to PspA and IM30, DynA aggregates to punctated foci *in vivo*, which are regions where membranes are supposed to be sealed.

Spatio-temporal TM defects result in locally increased proton concentrations at defined TM regions. Due to a locally decreased pH, the IM30 structure rearranges, resulting in an increased membrane-binding affinity. Thus, the spatio-temporal drop in the pH enhances preferential binding of IM30 to leaky membrane regions. The lowered pH also renders IM30 more fusion competent, and IM30 seals defect membrane regions, which involves membrane fusion processes.

## 4. Materials and Methods 

### 4.1. Cloning, Expression, and Purification of IM30

IM30 of *Synechocystis* sp. PCC 6803 was heterologously expressed in *Escherichia coli* BL21 DE3 and purified as described [11,13,21,37]. In short, after expression, cells were lysed via sonification in lysis buffer (50 mM Na-Phosphate, 20 mM imidazole, 300 mM NaCl pH 7.6). The lysate was cleared by centrifugation and the supernatant was applied to a Ni^2+^-NTA affinity column. The column was washed with increasing concentrations of imidazole and the protein was eluted with 500 mM imidazole. The elution fractions were pooled and the buffer was exchanged to 20 mM HEPES pH 7.6 by dialysis or gel-filtration (Sephadex G25).

### 4.2. Circular Dichroism Spectroscopy

Circular dichroism (CD) spectra were measured using a JASCO J815 CD spectrometer (JASCO Corporation, Tokyo, Japan). Spectra ranging from 190 to 260 nm were recorded at 20 °C with a scan rate of 100 nm/min, 1 nm steps and 1 s data integration time. Each sample contained 0.1 mg/mL IM30 and 10 mM HEPES buffer pH 7.6 or a mixture of HEPES buffer and phosphate buffer (pH 2.2) for each tested pH value in the range of pH 7.6 to 2.6. The samples were incubated for at least 15 min at room temperature. For each sample, three spectra were averaged and smoothed by the JASCO software package (Savitzky-Golay filter), if necessary. For quantification of the spectral change, the spectra were fitted with three gaussian peaks, using the Multi-PeakFit function of Origin2019 (OriginLabs), which rendered values for the center of the peak and the peak areas.

### 4.3. ANS Fluorescence Measurements

If not mentioned otherwise, all fluorescence measurements were performed using a FluoroMax-4 fluorimeter (Horiba Scientific, Kyoto, Japan) with an integration time of 0.1 s at 25 °C. For the ANS fluorescence measurements, 1 µM IM30 was mixed with 7.5 µM 8-anilinonaphthalene-1-sulfonic acid (ANS) (Sigma-Aldrich, Munich, Germany) in a mixture of 20 mM HEPES buffer (pH 7.6) and phosphate buffer (pH 2.2), resulting in samples with pH values ranging from pH 7.6 to 2.6. The samples were incubated in the dark for at least 15 min at room temperature. The excitation wavelength was 370 nm (slit widths 4 nm), the emission wavelength ranged from 400 to 650 nm (slit widths 4 nm). Fluorescence intensities of at least three independent measurements were averaged and spectra of ANS in the respective buffer in absence of the protein were subtracted.

### 4.4. Trp Fluorescence Measurements

1 µM IM30 were incubated in buffer (pH 2.6 – pH 7.6) for 2 h at 25 °C. Trp fluorescence was measured at 25 °C from 300 to 450 nm (slit width 3 nm) upon excitation at 280 nm (slit width 3 nm). The measurements were performed using a Fluoromax-4 spectrometer (Horiba Scientific, Kyoto, Japan).

### 4.5. Liposome Preparation

The lipids DOPG (1,2-dioleoyl-*sn*-glycero-3-phosphoglycerol), DOPC (1,2-dioleoyl-*sn*-glycero-3-phosphocholine), MGDG (monogalactosyldiacylglycerol) and the fluorescently labeled lipids NBD-PE (1,2-distearoyl-*sn*-glycero-3-phosphoethanolamine-N-(7-nitro-2-1,3-benzoxadiazol-4-yl) and LissRhod-PE (Lissamine Rhodamine PE; 1,2-Dioleoyl-*sn*-glycero-3-phosphoethanolamine-N-(Lissamine Rhodamine B sulfonyl) (ammonium salt)) were purchased from Avanti Polar Lipids, Inc. (Birmingham, AL, USA). For liposome preparation, lipids were dissolved in chloroform/methanol (2:1, v/v). The organic solvent was evaporated under a gentle stream of nitrogen gas followed by overnight vacuum desiccation to remove any traces of solvent. Unilamellar liposomes were prepared by rehydration of the dried lipid film with the corresponding buffer and five cycles of freeze-thawing.

For use in QCM measurements, the rehydrated lipid film was subsequently sonified with a tip-sonicator in an ice bath followed by centrifugation (10 min 16,500 g) to produce smaller liposomes. For the liposome fusion assay, the liposomes were instead extruded 15 times through a 100 nm filter, using an extruder from Avanti Polar Lipids, Inc. (Alabaster, AL, USA). 

### 4.6. Laurdan Fluorescence Measurements

Laurdan (6-dodecanoyl-*N*,*N*-dimethyl-2-naphthylamine) is a fluorescent dye that incorporates into lipid bilayers. Its fluorescence is sensitive to changes in the polarity of the environment and is therefore used to report changes of the membrane fluidity. In order to quantify the spectral changes, the Generalized Polarization (GP) value defined by [71] is calculated for each spectrum.

(1) $$\mathrm{GP}=\frac{I_{440}{-I}_{490}}{I_{440}{+I}_{490}}$$

Here, I_440_ and I_490_ are the fluorescence emission intensities at 440 and 490 nm, respectively.

Laurdan (Sigma, Taufkirchen, Germany) was added to the dissolved lipids MGDG/DOPG (40:60, w/w) in a molar ratio of 1:500. Unilamellar liposomes were prepared as described before. To analyze the effect of acidic pH on binding of IM30 to the liposomes, 0 - 6 µM IM30 and 0.1 mM liposomes in the corresponding buffer were mixed and incubated for 30 min in the dark at 25 °C. Buffers with different pH were prepared by mixing appropriate portions of 20 mM Hepes (pH 7.6) and 50 mM sodium acetate buffer (pH 4.8).

The fluorescence emission spectra were recorded at 25 °C on an FP8500 (JASCO Corporation, Tokyo, Japan) from 400 to 600 nm with excitation at 350 nm. The slit width was set at 2.5 nm for excitation and emission of Laurdan.

### 4.7. Membrane Fusion Assay

The influence of acidic pH on IM30-triggered liposome fusion was measured using a FRET-based assay, as described earlier [21,72]. Unlabeled liposomes were mixed in 10-fold excess with labeled liposomes containing two fluorescent dyes that form a FRET pair. Upon fusion of labeled with unlabeled liposomes, the FRET dyes redistribute over the membrane and consequently FRET decreases, resulting in increasing donor emission intensity. To simulate complete membrane fusion, liposomes containing only 1/10 of the fluorescently labeled lipids were used as a positive control.

To analyze the spontaneous fusion of MGDG/DOPG (40:60, w/w) membranes in absence of IM30, membrane fusion was induced via addition of 15 mM Mg^2+^. For the IM30-dependent membrane fusion assay, 2.5 µM IM30, 0.1 mM liposomes (MGDG/DOPG, 40:60, w/w) and 7.5 mM Mg^2+^ were used. All components were dissolved in an appropriate mixture of 20 mM Hepes (pH 7.6) and 50 mM sodium acetate buffer (pH 4.8).

The measurements were performed using a Fluoromax-4 spectrometer (Horiba Scientific, Kyoto, Japan). The IM30-containing solutions were preincubated with Mg^2+^ for about 5 min. After fast mixing of all compounds, the measurement was started immediately. Upon excitation of the FRET-donor NBD-PE at 460 nm, the donor emission was monitored at 525 nm over 300 s at 25 °C. The slit widths for excitation and emission were set at 5 nm.

The raw fluorescence data was converted to a fusion rate by equation 2, using the intensities of the negative control (*I_0_*), positive control (*I_M_*) and the measured sample (*I*) at every point in time *t*.

(2) $$\mathrm{NBD}\;\left(t\right)=\frac{{I-I}_{0}}{I_{M}{-I}_{0}}$$

The initial fusion rate was determined by the slope of the fusion curve over the first 50 s.

### 4.8. Quartz Crystal Microbalance

For QCM measurements, only degassed buffers were used. QCM chips were cleaned with 30 mM EDTA, 2% SDS followed by 1 M NaOH. Then the chip was rinsed with water and dried with nitrogen. Prior to the measurement, the chip was treated with an ozone plasma cleaner for 20 s to remove any organic contaminations. Fifty microliters of a liposome suspension (1 mM lipid, 80% DOPC 20% DOPG (w/w)) was mixed with 450 µL 20 mM HEPES + 5 mM CaCl_2_. A SiO_2_-coated QCM chip (3T Analytik, Tuttlingen, Germany) was calibrated with the QCM device (3T Analytik, Tuttlingen, Germany). To produce an SLB on the SiO_2_ surface, the chip was washed with HEPES buffer. Next, 150 µL of the liposome suspension was pumped on the chip (60 µL/min). After completion of liposome spreading (i.e., when the frequency shift reached a constant level with a damping signal close to zero), the chip was washed again with HEPES buffer (pH 7.6) or HEPES/acetate buffer (pH 5.5) (150+300 µL; 60 µL/min). To start the measurement, 150 µL IM30 wt (4.5 µM in HEPES buffer or HEPES/acetate buffer was pumped on the SLB (60 µL/min). The pump was stopped and binding of the protein was monitored over ~3500 s. 

## Supplementary Materials

Supplementary materials can be found at https://www.mdpi.com/1422-0067/21/12/4530/s1.

## Abbreviations

IM30	Inner-membrane associated protein of 30 kDa
PspA	Phage shock protein A
PMF	Proton motive force
TM	Thylakoid Membrane
CD	Circular Dichroism
SD	Standard Deviation
QCM	Quartz Crystal Microbalance
