# Supplementary material for: Proton Leakage Is Sensed by IM30 and Activates IM30-Triggered Membrane Fusion
Source: Int J Mol Sci. 2020 Jun 25;21(12):4530. doi: 10.3390/ijms21124530 (PMC7350238; doi:10.3390/ijms21124530)
Supplement: Supplementary file 1 [file ijms-21-04530-s001.pdf]

Supplemental Figures

Proton Leakage is Sensed by IM30

and Activates IM30-Triggered Membrane fusion

Carmen Siebenaller[#], Benedikt Junglas[#], Annika Lehmann, Nadja Hellmann and Dirk Schneider[*]

Department of Chemistry, Membrane Biochemistry, Johannes Gutenberg University Mainz, 55128 Mainz, Germany.

[#]These authors contributed equally

***Address correspondence to**

Dirk Schneider, Johannes Gutenberg University Mainz, Department of Chemistry, Johann-Joachim-Becher-Weg 30, 55128 Mainz, Germany

Phone: (+49) 6131 39-25833, Fax: (+49) 6131 39-25348, E-mail: Dirk.Schneider@uni-mainz.de

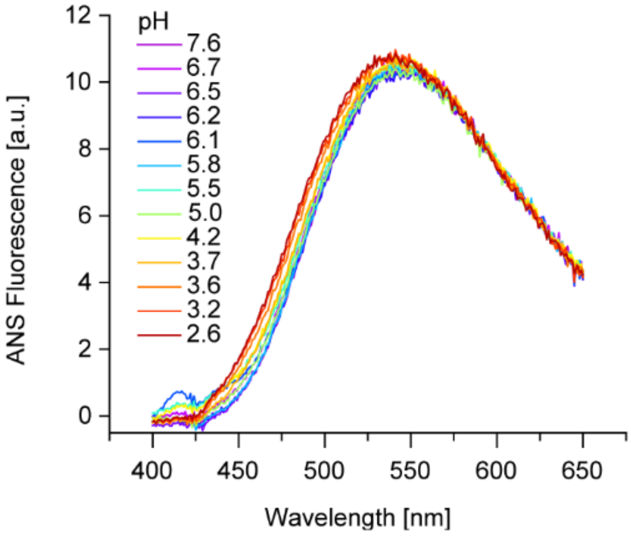

Suppl. Figure S1: ANS fluorescence is not affected by low pH. The fluorescence of the ANS fluorophore was measured at pH values ranging from pH 7.6 to 2.6. Besides a minor blue shift, no influence of the pH on the general fluorescence properties was observed. The spectra are averages from 3 samples. No error bars are shown.

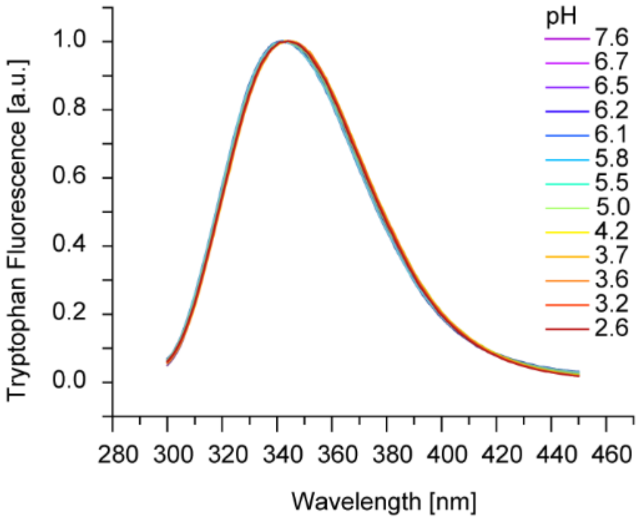

Suppl. Figure S2: Complete Trp fluorescence spectra. Intrinsic Trp fluorescence spectra of IM30 were measured from 300 to 450 nm. Detailed analysis is presented in Figure 2c. The spectra are averages of three samples. No error bars are shown.

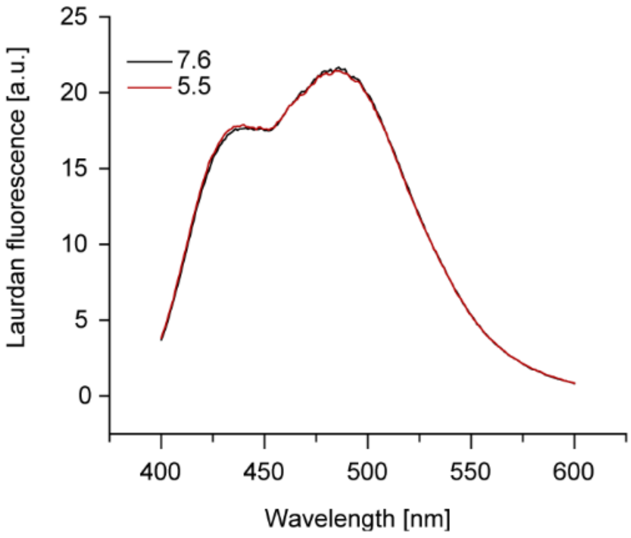

Suppl. Figure S3: Laurdan fluorescence is not influenced by pH. The fluorescence of Laurdan in DOPG/MGDG (40:60) liposomes was measured at pH 7.6 and 5.5. No change of the Laurdan fluorescence has been observed. The spectra of 3 samples were averaged. No error bars are shown.

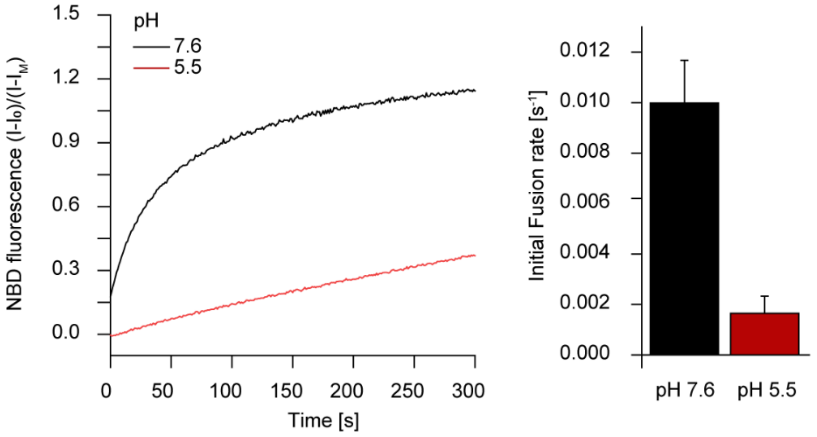

Suppl. Figure S4: Mg^{2+}-triggered membrane fusion is diminished at pH 5.5. (a) Liposome fusion was measured in the presence of 15 mM Mg^{2+} by a FRET-based fusion assay at pH 5.5 (black) and pH 7.6 (red). At pH 5.5, a strongly decreased membrane fusion was observed compared to membrane fusion at pH 7.6. The curves represent the average of 3 independent measurements. No error bars are shown. The initial fusion rates (b) were calculated over the first 50 s (n=3, error bars=SD).
